# Integrated Multi-Omics Data Analysis Reveals Associations Between Glycosylation and Stemness in Hepatocellular Carcinoma

**DOI:** 10.3389/fonc.2022.913432

**Published:** 2022-06-23

**Authors:** Peiyan Liu, Qi Zhou, Jia Li

**Affiliations:** ^1^ Department of Hepatology, Second People’s Clinical College of Tianjin Medical University, Tianjin, China; ^2^ Department of Hepatology, Tianjin Second People’s Hospital, Tianjin, China; ^3^ Department of Gastroenterology, The Third Affiliated Hospital of Sun Yat-Sen University, Guangzhou, China; ^4^ Guangdong Provincial Key Laboratory of Liver Disease Research, Guangzhou, China

**Keywords:** hepatocellular carcinoma, glycosylation, stemness, copy number variations, immunity, prognosis, CAD

## Abstract

**Background:**

Glycosylation plays an essential role in driving the progression and treatment resistance of hepatocellular carcinoma (HCC). However, its function in regulating the acquisition and maintenance of the cancer stemness-like phenotype in HCC remains largely unknown. There is also very little known about how CAD and other potential glycosylation regulators may influence stemness. This study explores the relationship between glycosylation and stemness in HCC.

**Methods:**

Gene set variance analysis (GSVA) was used to assess the TCGA pan-cancer enrichment in glycosylation-related pathways. Univariate, LASSO, and multivariate COX regression were then used to identify prognostic genes in the TCGA-LIHC and construct a prognostic signature. HCC patients were classified into high- and low-risk subgroups based on the signature. The relationship between gene expression profiles and stemness was confirmed using bulk and single-cell RNA-sequencing data. The role of CAD and other genes in regulating the stemness of HCC was also validated by RT-qPCR, CCK-8, and colony formation assay. Copy number variation (CNV), immune infiltration, and clinical features were further analyzed in different subgroups and subsequent gene expression profiles. Sensitive drugs were also screened.

**Results:**

In the pan-cancer analysis, HCC was shown to have specific glycosylation alterations. Five genes, CAD, SLC51B, LGALS3, B3GAT3, and MT3, identified from 572 glycosylation-related genes, were used to construct a gene signature and predict HCC patient survival in the TCGA cohort. The results demonstrated a significant positive correlation between patients in the high-risk group and both elevated gene expression and HCC dedifferentiation status. A significant reduction in the stemness-related markers, CD24, CD44, CD20, FOXM1, and EpCAM, was found after the knockdown of CAD and other genes in HepG2 and Huh7 cells. Frequent mutations increased CNVs, immune-suppressive responses, and poor prognosis were also associated with the high-risk profile. The ICGC-LIRI-JP cohort confirmed a similar relationship between glycosylation-related subtypes and stemness. Finally, 84 sensitive drugs were screened for abnormal glycosylation of HCC, and carfilzomib was most highly correlated with CAD.

**Conclusions:**

Glycosylation-related molecular subtypes are associated with HCC stemness and disease prognosis. These results provide new directions for further research on the relationship between glycosylation and stemness phenotypes.

## Introduction

Hepatocellular carcinoma (HCC) accounts for 75–85% of primary liver cancers and is the second leading cause of cancer death, with 5-year survival rates of only 4–17% (1). This disease is highly malignant and progresses rapidly, resulting in almost one million deaths each year (2). Surgical resection of HCC followed by chemotherapy is an ideal curative treatment strategy, but it is limited by advanced stage and metastasized tumor cells. While several new therapeutic agents, including checkpoint or tyrosine kinase inhibitors, have been approved by the FDA for patients who cannot undergo surgery or transplantation, their efficacy is unsatisfactory (3, 4), and HCC patients still have an average survival of only 6 months (5, 6). Liver cancer stem cells (LCSCs) are closely associated with the poor prognosis of HCC because they have more robust metastatic and tumorigenic properties than non-LCSCs. Thus, eliminating LCSCs or reducing tumor size is critical to improving HCC treatment efficacy (7). There is an urgent need to explore the molecular biological features of LCSCs.

Glycosylation is a complex process by which a carbohydrate is added to a protein or lipid carrier and is involved in many cellular mechanisms, including cell–cell adhesion, trafficking, motility, inflammation, signaling, host–pathogen interactions, and innate immune responses. All these processes play an essential role in the development and progression of HCC (8, 9). Aberrant glycosylation is frequently cited as a hallmark of malignancy, and from the perspective of epigenetics, glycosylation is thought to directly impact key processes supporting the stemness of HCC, including cell adhesion, motility, invasion, and evasion (9–11). With the presence of highly expressed glycosyltransferases, altered glycosylation is ubiquitous in HCC cells.

Carbamoyl-phosphate synthetase 2, aspartate transcarbamoylase, and dihydrooro-tase (CAD) are multifunctional proteins that play prominent roles in glycosylation. CAD mutations can significantly reduce glycosylation and angiogenesis (12). A product of CAD, uridine diphosphate (UDP), is a specific target for interventional tumors (13, 14). Numerous drugs and anti-tumor vaccines targeting glycosylation are currently in clinical trials. One drug, trastuzumab, is shown to increase the sensitivity of drug-resistant breast cancer cell lines by removing siglec ligands and boosting antibody-dependent natural killer (NK) cell cytotoxicity (15). More glycomic and glycoproteomic studies will help define novel targets and strategies for improving cancer treatment (16). While the B3GAT3, SLC51B, LGALS3, and MT3 genes also play an essential role in the glycosylation pathway, their relationship with stemness remains unclear.

Because of the Warburg effect, sustained high glucose levels in HCC can promote abnormal glycosylation reactions, activate particular signaling pathways, and produce irreversible toxic products, such as glyoxal, methylglyoxal, and 3-deoxyglucosone, that accelerate HCC proliferation and metastasis (17–20). Glycosylation of the stem cell markers, CD24, CD20, CD44, EpCAM, and FOXM1, plays an important role in regulating LCSCs (21–23). Tumor-related glycoprotein or glycan antigen alterations approved by the Food and Drug Administration (FDA), such as core fucosylated AFP (AFP-L3), are better targets for tumor diagnosis and prognosis than AFP alone (24). Further understanding of HCC-related glycosylation patterns will provide advances in treatment and prognosis and reduce mortality.

In this study, glycosylation-related gene sets were downloaded from the GSEA (http://www.gsea-msigdb.org) and a model of HCC prognosis was constructed. Bulk and single-cell RNA-sequencing revealed that the genes and model correlated closely with the stemness of HCC. A systematic analysis of the multi-omics results, including CNV mutations, transcription factors, immunity, and clinical characteristics, may inform further study of the relationships between glycosylation and the stemness of HCC.

## Materials and Methods

### Pan-Cancer Data Collection and Analysis

All glycosylation-related pathways and relevant gene sets were retrieved from the Molecular Signatures Database (MSigDB, http://www.gsea-msigdb.org/gsea/msigdb/) (**Table S1**). TCGA pan-cancer RNA-seq data (FPKM values) were downloaded from the genomic data common website (https://gdc.cancer.gov/about‐data/publications/pancanatlas). Gene set variance analysis (GSVA) was used to assess the enrichment of each pan-cancer sample in glycosylation-related pathways, and the distribution of scores was shown using the R “pheatmap” package. Principal component analysis (PCA) was performed after scaling by Z-score, and the pan-cancer RNA-seq data were projected into two dimensions.

### HCC Data Collection

In total, 424 RNA-seq transcriptome cases with corresponding clinical information were extracted from the TCGA-LIHC database (https://portal.gdc.cancer.gov) using the Genomic Data Commons (GDC) tool. RNA-seq data were normalized by FPKM. The somatic mutation and CNV data were also downloaded from the TCGA. RNA-seq and clinicopathological data from a Japanese HCC cohort were obtained from the ICGC (LIRI-JP, https://dcc.icgc.org/projects/LIRI-JP) as a comparison.

### Construction of a Potential Prognostic Signature

First, 143 differentially expressed genes (DEGs) were identified using LIMMA analysis (adjusted *P <*0.05 and |Log FC| >1). Univariate Cox regression analysis was then performed to identify potential prognostic DEGs (*P <*0.05). Least absolute shrinkage and selection operator (LASSO) regression analysis and stepwise Cox regression analysis were used to construct a prognostic model. The risk score of each sample was calculated using the following formula:


$$\text{Risk score}=\;\sum_{i}^{k}Xi\times Yi\left(X:coefficients,\;Y:gene\;expression\;level\right).$$

Based on risk scores, the samples were divided into high- and low-risk subgroups.

### Calculation of Stemness-Associated Scores

The mRNA-si stemness score (25) and other stemness signatures (Ben-Porath ESC score (26), Wong ESC score (27), and Bhattacharya ESC score (28)) were used to assess stemness in both the TCGA-LIHC and ICGC-LIRI-JP cohorts.

### Single-Cell RNA-Sequencing Data Analysis

Smart-seq2 data (GSE103866) from 55 HuH-1 cells, 63 HuH-7 cells, and 12 patient HCC cells were obtained from the Gene Expression Omnibus (GEO) website. After preprocessing, the scRNA-seq data were converted into a Seurat object, and further quality control was conducted. Data were excluded if 1) cells had >30% mitochondrial genes, 2) genes were identified in <3 cells, or 3) cells had <300 detectable genes. Log normalization, centralization, generation of hypervariable genes, PCA, and clustering analysis were used to perform dimensionality reduction. Uniform manifold approximation and projection (UMAP) was used to visualize the cell distribution using EpCAM, CD24, and CD133 as cell surface markers. “FeaturePlot” and “VlnPlot” were also used to visualize the glycosylation-related genes.

### Experimental Validation of the Relationship Between Gene Expressions and Stemness Phenotype

The expression of the HepG2, PLC, Huh7, and Hep3B genes in HCC tumors and cells was determined by quantitative real-time PCR (RT-qPCR) according to the protocols of the manufacturer. Cell Counting Kit-8 (CCK-8) and colony formation assays were used to measure cell proliferation. Small interfering RNA (siRNA) was transfected using Lipofectamine 2000 (Invitrogen, Carlsbad, CA, USA) and mRNA levels were assessed after 48 h. The primer and specific siRNA sequences are included in **Table S2**.

### Analysis of Somatic Mutations and Gene Copy Number Variations (CNVs)

Somatic mutation and CNV data were extracted from the TCGA. The somatic mutation data were visualized using the R “maftools” package. GISTIC2.0 was used to determine the significantly deleted or amplified genomic regions in low- and high-risk subgroups in TCGA-LIHC, referenced to the Consortium Human build 38 (GRCh38). Gene locations were obtained online (ftp://ftp.ensembl.org/pub/current_gtf).

### Analysis of Tumor Immune Infiltration

Six previously reported immune subtypes of TCGA-LIHC were identified (29). Infiltrating immune cell fractions were analyzed in tumor samples using single-sample gene set enrichment analysis (ssGSEA) and cell-type identification. Relative subsets of RNA transcript (CIBERSORT) algorithms were estimated in both the TCGA-LIHC and ICGC-LIRI-JP cohorts. Intratumor heterogeneity, IFN-response, TGF-β response, proliferation, and wound healing scores were estimated in the low- and high-risk groups (29).

### Analysis of Clinical Data

Kaplan–Meier survival curves were used to evaluate the overall survival (OS) of risk subgroups from the TCGA-LIHC and ICGC-LIRI-JP cohorts. The predictive sensitivity and specificity of the gene signatures were assessed by receiver operating characteristic (ROC). PCA was conducted using the R “prcomp” function of the “stats” package. Univariate and multivariate Cox regression analyses were performed to identify whether clinical characteristics and risk scores were independent risk factors. A nomogram was established to predict the 1-, 3-, and 5-year OS using the R “rms” package. Calibration plots, concordance index (C-index), and ROC were used to evaluate nomogram performance.

### Analysis of Drug Sensitivity

The drug sensitivity inhibitory concentration (IC50) value and the mRNA profiles of NCI60 cell lines were extracted from the Cell-Miner™ database (https://discover.nci.nih.gov/cellminer/home.do). The sensitivity of the five selected genes was tested against 216 FDA-approved drugs. Co-expression network analysis of the genes and drugs was visualized using Cytoscape.

## Results

### HCC Has Specific Glycosylation Alterations in Human Pan-Cancer

**Figure 1:**
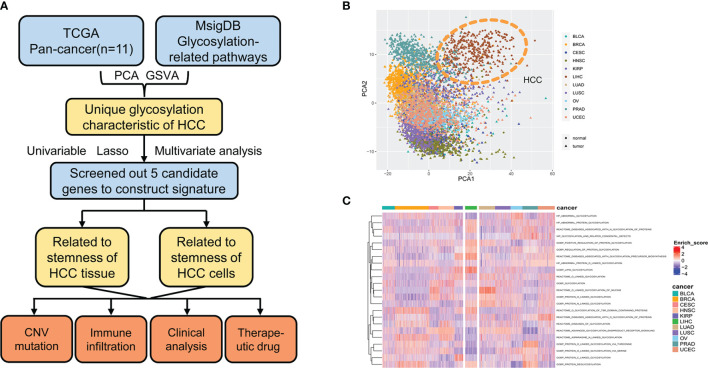
Glycosylation alterations in human pan-cancer. **(A)** Study flowchart. **(B)** PCA projection of paired tumor and normal tissue samples from 11 different cancer types in TCGA. Different colors represent different cancer types. Circles and triangles represent normal and tumor tissue, respectively. **(C)** Heatmap showing glycosylation-related pathway alterations across 11 cancer types. MsigDB, molecular signatures database; PCA, principal component analysis; HCC, hepatocellular carcinoma; GSVA, gene set variance analysis; CNV, copy number variation; BLCA, bladder urothelial carcinoma; BRCA, breast invasive carcinoma; CESC, cervical squamous cell carcinoma and endocervical adenocarcinoma; HNSC, head and neck squamous cell carcinoma; KIRP, kidney renal papillary cell carcinoma; LIHC, liver hepatocellular carcinoma; LUAD, lung adenocarcinoma; LUSC, lung squamous cell carcinoma; OV, ovarian serous cystadenocarcinoma; PRAD, prostate adenocarcinoma; UCEC, uterine corpus endometrial carcinoma.

A flowchart of this study is shown in **Figure 1A**. mRNA expression of glycosylation-related genes across 11 cancer types in TCGA was evaluated using PCA. HCC had the most distinct glycosylation patterns (**Figure 1B**). The GSVA value of glycosylation-related pathways was also calculated in different samples of 11 pan-cancers, and HCC had the most specific enrichment of all cancers (**Figure 1C**).

### Identification of the Candidate Genes and Construction of the Prognostic Signature of HCC

**Figure 2:**
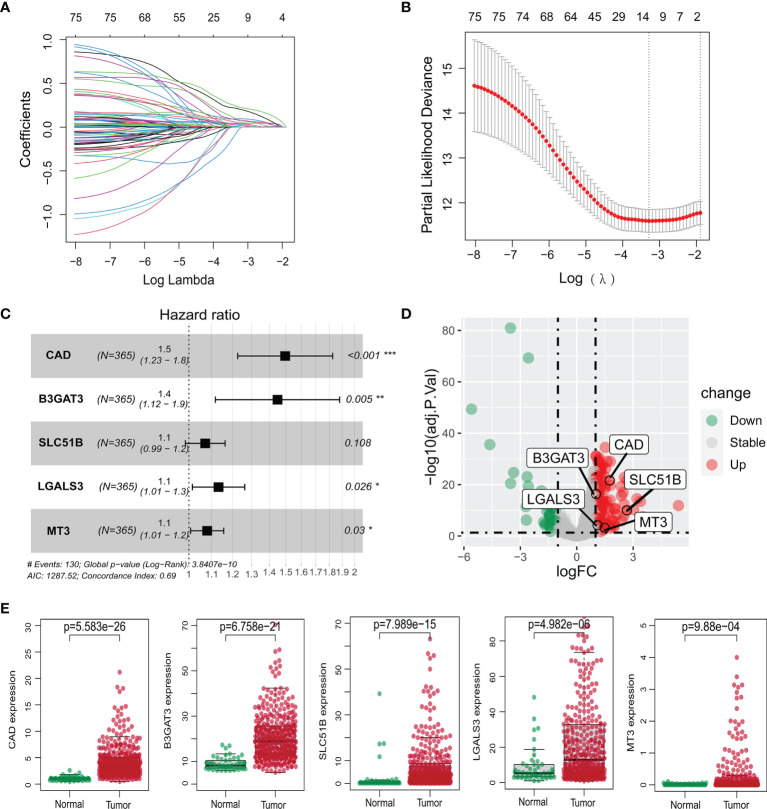
Construction of the prognostic model of the TCGA-LIHC cohort. **(A, B)** Lasso regression analysis of survival-associated genes. **(C)** Multivariate Cox regression confirming five glycosylation-related genes. **(D)** Volcano plot of differentially expressed genes between HCC and non-tumor tissues. The five genes are marked. Red: significant upregulation; blue: significant downregulation; grey: no statistical significance. **(E)** Expression levels of five glycosylation-related genes between normal and HCC samples. *P <0.05; **P <0.01; *** *P <*0.001.

Using gene expression profiling and corresponding clinical information from 50 normal and 374 tumor samples in the TCGA-LIHC database, 143 glycosylation-related DEGs were selected (adjusted *P <*0.05 and |Log FC| >1). Univariate Cox regression, LASSO regression, and stepwise Cox regression analyses were used to further investigate the importance of these DEGs (**Figures 2A, B**; **Table 1**). Five glycosylation-related genes were selected to construct the prognostic signature. Risk score = (0.403 ∗ CAD exp.) + (0.371 ∗ B3GAT3 exp.) + (0.068 ∗ SLC51B exp.) + (0.124 ∗ LGALS3 exp.) + (0.0767 ∗ MT3 exp.).

**Table 1:** Multivariate Cox analysis results of glycosylation-related genes.

Id	Coef	HR	HR.95L	HR.95H	*P-*value
CAD	0.402886	1.496136	1.226555	1.824968	7.05E−5
B3GAT3	0.370858	1.448978	1.117225	1.879242	0.005181
SLC51B	0.068264	1.070648	0.9851	1.163626	0.108134
LGALS3	0.12411	1.13214	1.014627	1.263264	0.026941
MT3	0.076625	1.079637	1.007436	1.157013	0.023701

HR, hazard ratio; L, low; H, high.

Based on the median risk score, HCC patients from the TCGA-LIHC and ICGC-LIRI-JP cohorts were classified into high- and low-risk groups. The five selected genes were identified as risk factors and had higher mRNA expressions in tumor tissues (**Figures 2C–E**). The protein expression patterns are presented in **Supplementary Figure 1**.

### Gene Expression Profiles of High-Risk Patients Were Enriched With HCC Stemness Markers in TCGC-LIHC and ICGC-LIRI-JP Cohorts

**Figure 3:**
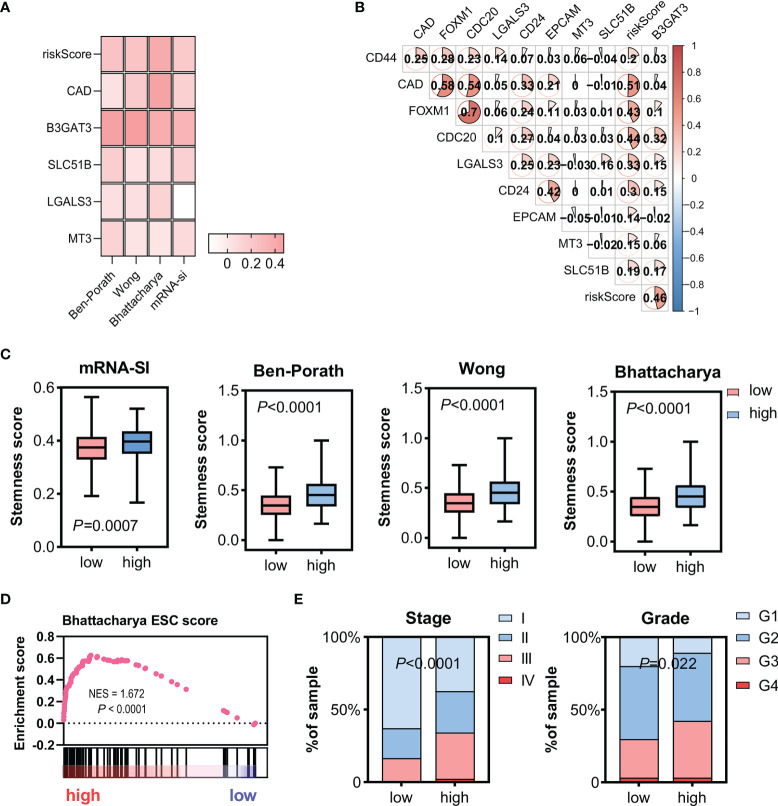
Relationship between gene expression profiles and HCC stemness using HCC bulk data from TCGA-LIHC. **(A)** Heatmap of Spearman’s correlation results of the gene expression profiles and four distinct stemness indices (Ben-Porath signature, Wong signature, Bhattacharya signature, and mRNA-si). A darker color represents a stronger correlation. **(B)** Correlation between the CSC markers, CD24, CD44, CD20, FOXM1, and EpCAM, and the gene expression profiles. **(C)** Different scores of the four distinct stemness indices between the low- and high-risk groups. **(D)** The transcriptome profiles of high-risk HCC patients were significantly enriched with stemness markers. **(E)** Among the high-risk patients, the frequency of higher stages and grades was more significantly elevated. Tumor stages and grades were color-coded as shown in the legend. NES, normalized enrichment score; CSC, cancer stem cells.

As reported previously, glycosylation is highly correlated with stemness. This study aimed to compare gene expression profiles and risk scores with stemness markers. A mostly positive Spearman’s correlation with the stemness-associated transcriptome-based signatures (Ben-Porath, Wong, Bhattacharya, and mRNA-si) was observed in the TCGA-LIHC (**Figure 3A**) and ICGC-LIRI-JP cohorts (**Supplementary Figure 2A**). The stemness markers, CD24, CD44, CD20, FOXM1, and EpCAM, also correlated strongly with gene expression levels and risk scores in both the TCGA-LIHC (**Figure 3B**) and ICGC-LIRI-JP cohorts (**Supplementary Figure 2B**). The high-risk cohorts had significantly higher signature scores (**Figure 3C**; **Supplementary Figure 2C**) and the gene expression profiles in the TCGA-LIHC and ICGC-LIRI-JP high-risk subgroups were significantly enriched in the Bhattacharya ESC signature (**Figure 3D**; **Supplementary Figure 2D**). The TCGA-LIHC and ICGC-LIRI-JP high-risk groups also had a significantly larger proportion of higher stages and grades (**Figure 3E**; **Supplementary Figure 2E**), suggesting the presence of a dedifferentiated phenotype.

### Expression Profiles of the Five Glycosylation-Related Genes Were Associated With Stemness Markers in LCSCs

**Figure 4:**
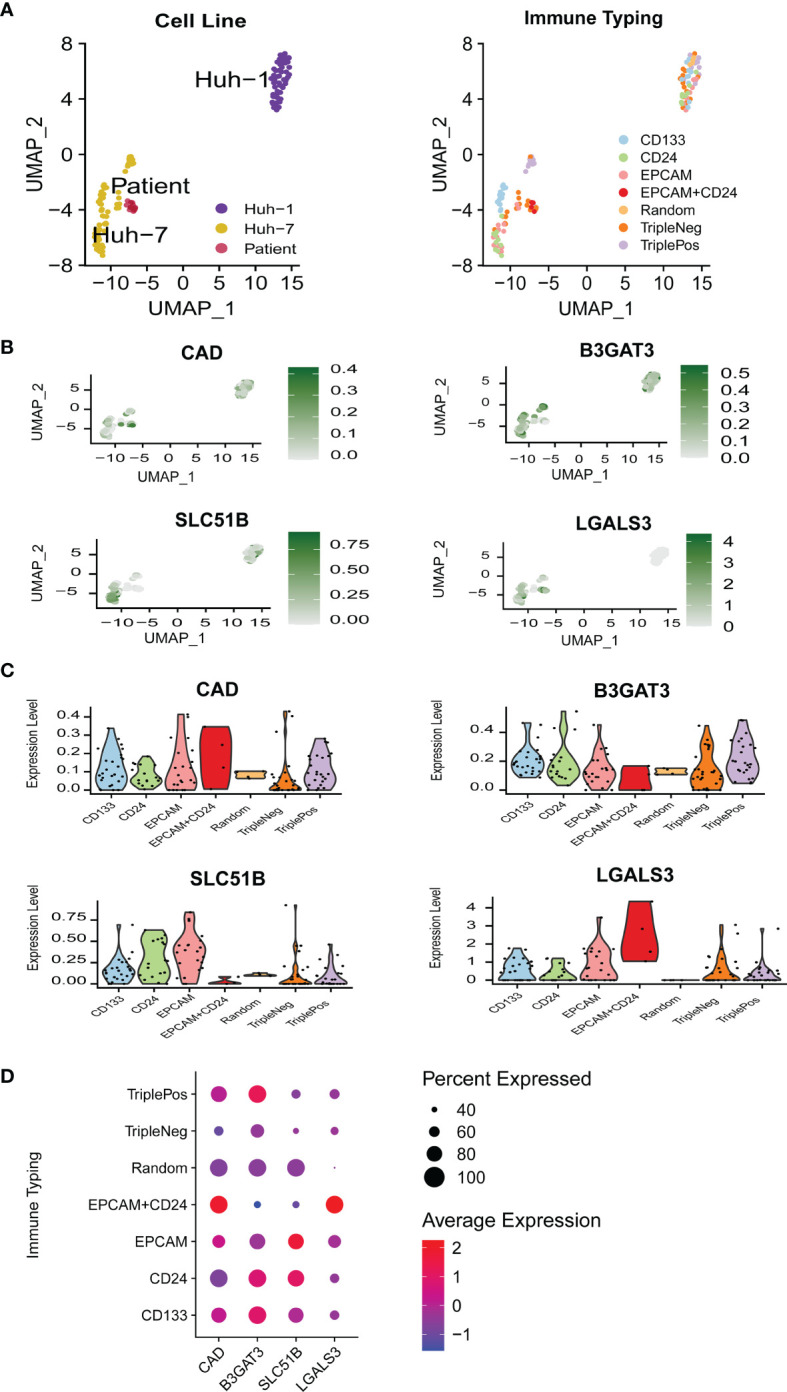
The relationship between gene expression profiles and HCC stemness in single-cell RNA-sequencing data. **(A)** UMAP plots of 130 single cells using different classifications. **(B)** UMAP plots of the four glycosylation-related genes in CSC (MT3 was not detected in GSE 103866). **(C)** Violin plots of the four glycosylation-related gene expressions in CSC. **(D)** Expression of the four glycosylation-related genes in different immune phenotypes. CSC, cancer stem cells.

To further define the relationship between gene expression profiles and stemness in terms of stem cells, a single-cell RNA-sequencing dataset (GSE103866) including 55 HuH-1 cells, 63 HuH-7 cells, and 12 patient-derived cancer stem cells (CSCs) was downloaded. The UMAP algorithm was adopted for the three cell types, three CSC markers, and the four gene expression distributions (**Figures 4A, B**). Vlnplot was used to visualize differences in marker gene expression in the distinct immunophenotypes (**Figure 4C**). Compared with the triple-negative CSCs, these genes showed high expression in other groups with stemness phenotypes (**Figure 4D**). These results confirm that high expression of these glycosylation-related genes may enhance the stemness of HCC.

### Experimental Validation of the Relationship Between the Five Glycosylation-Related Genes and Stemness

**Figure 5:**
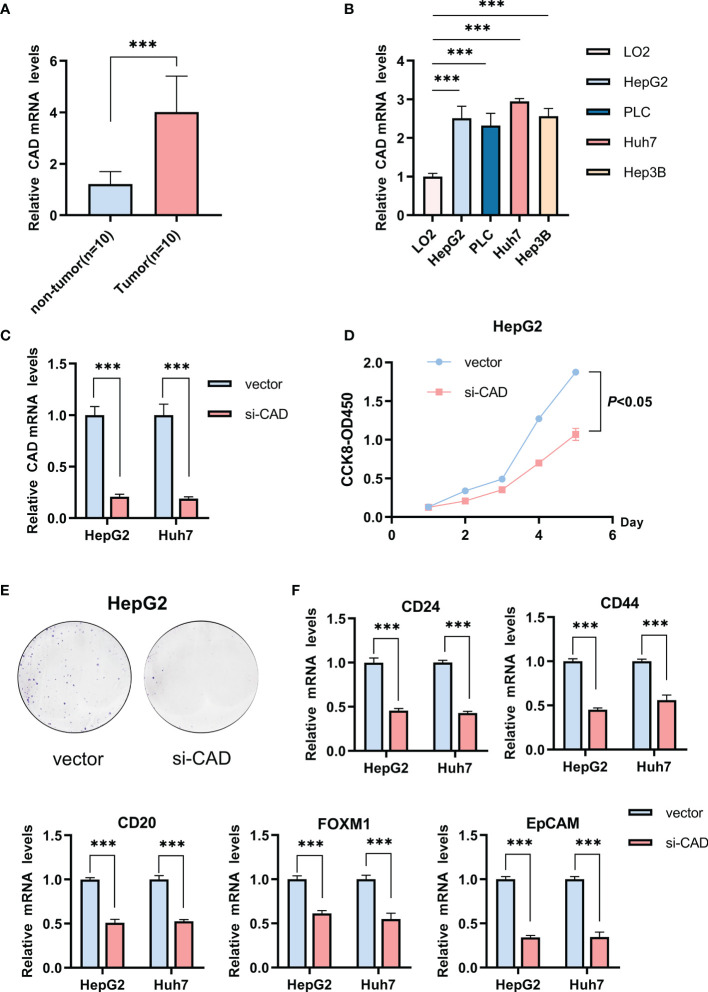
The influence of CAD on the HCC stemness phenotype. **(A)** Differences in CAD expression between normal and HCC tissues. **(B)** Differential expression of CAD in HCC and normal cells. **(C)** The efficiency of CAD knockdown in HepG2 and Huh7 cells. **(D)** CCK-8 experiment comparing the si-CAD and control groups in HepG2 cells. **(E)** Colony formation assay comparing the si-CAD and control group in HepG2 cells. **(F)** Significant decrease in stemness-related markers, CD24, CD44, CD20, FOXM1, and EpCAM, after CAD knockdown in HepG2 and Huh7 cells. ***P <0.001.

The relationship between the five genes and stemness was also verified in HCC samples. CAD expression was the most different between HCC and normal tissues in the TCGA-LIHC cohort. HCC patient tissues and cell lines also had significantly higher CAD mRNA levels than normal samples (**Figures 5A, B**). Gene knockdown was conducted in the HepG2 and Huh7 cell lines and the efficiencies were verified (**Figure 5C**). CAD knockdown resulted in a significant decline in tumor cell viability (*P <*0.05; **Figure 5D**) and a marked decrease in the proliferative capacity (**Figure 5E**). CD24, CD44, CD20, FOXM1, and EpCAM expression were also significantly lower in both si-CAD HepG2 and Huh7 cells than in control cells (**Figure 5F**). Knockdown of the remaining four genes also significantly inhibited CD24, CD44, CD20, FOXM1, and EpCAM expression (**Supplementary Figure 3**).

### Somatic Mutation Alterations and CNVs in Different Gene Expression Profiles

**Figure 6:**
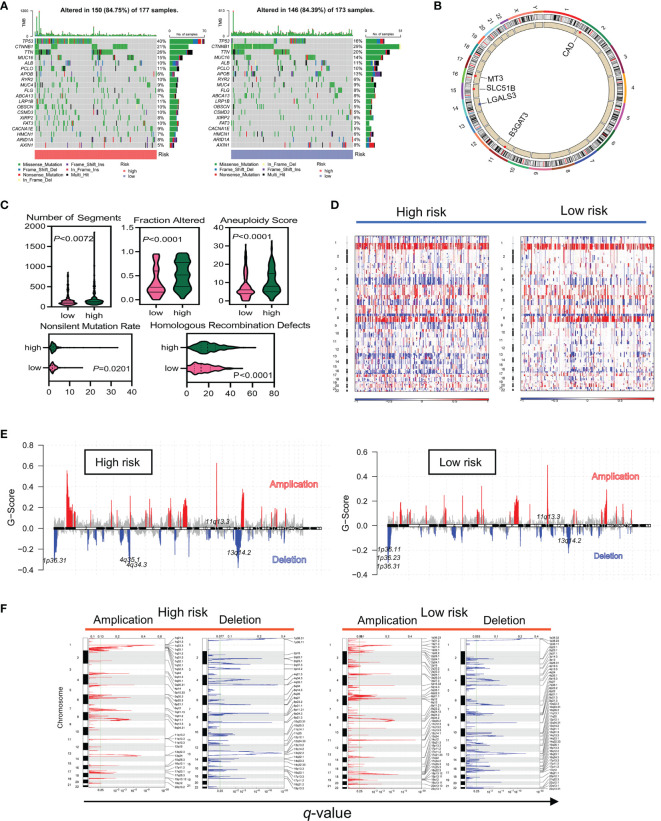
The high-risk group had a higher frequency of somatic mutations and CNVs in TCGA data. **(A)** Differences in somatic mutation frequency in the high- and low-risk groups. **(B)** Five gene copy loss and copy amplification proportion distributions in the genome. **(C)** Scores of the five distinct mutation indices (number of segments, fraction altered, aneuploidy score, nonslinet mutation rate, and homologous recombination defects) in the low- and high-risk groups. **(D–F)** CNVs in different risk subgroups. Red and blue represent the two types of CNVs, amplification and deletion, respectively.

Gene mutations in normal stem or progenitor cells may lead to the development and activation of LCSCs and are closely linked to the stemness of HCC. In mutation frequency analyses, a waterfall diagram showed the different status of somatic mutations in the TCGA-LIHC low- and high-risk groups. Sixty percent of genes, including TP53, TTN, MUC16, RYR2, LRP1B, OBSCN, CSMD3, XIRP2, FAT3, CACNA1E, HMCN1, and ARID1A, had a higher mutation frequency in the high-risk than in the low-risk group, while only 20%, including CTNNB1, APOB, ALB, and AXIN1, had a higher frequency in the low-risk group (**Figure 6A**). The chromosomal locations and CNV alterations of the glycosylation-related genes are shown in **Figure 6B**. High-risk patients also had a higher number of segments and some mutation scores (29) (**Figure 6C**). Many chromosomal regions showed significant copy amplification and gene deletion. CNVs were associated with a high-risk prognosis (**Figure 6D**). GISTIC showed that the scores of both amplification and deletion CNVs were considerably higher in the high-risk group than in the low-risk group (**Figure 6E**). While the low-risk group had a higher frequency of CNVs than the high-risk group, the high-risk group had more variability (**Figure 6F**).

### The Landscape of Immune Infiltration in Different Gene Expression Profiles

**Figure 7:**
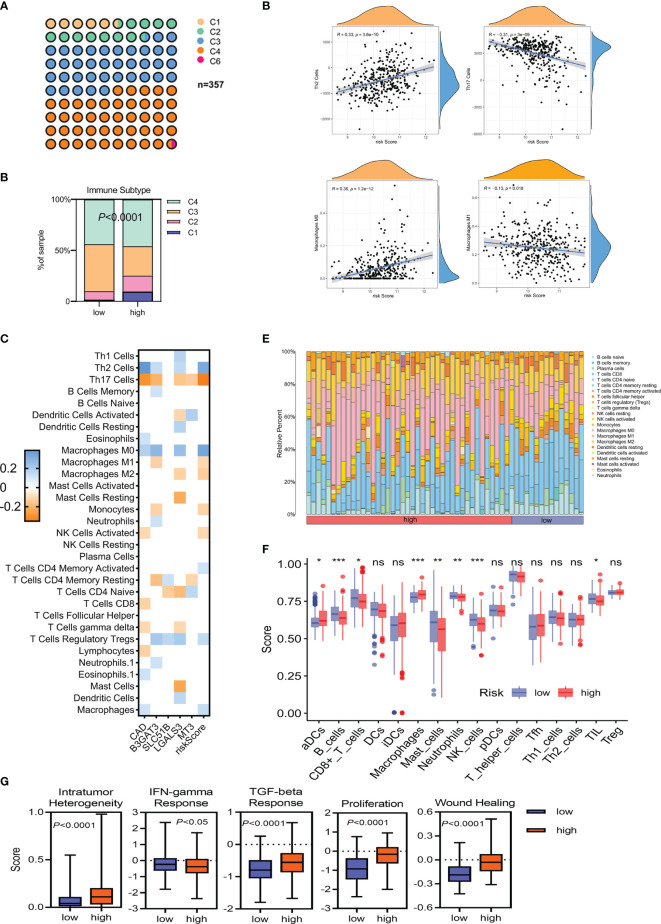
The immune landscape of HCC tumors in TCGA-LIHC. **(A, B)** Classification of low- and high-risk HCC tumors into C1–C6 classes. C1, wound healing; C2, IFN-γ dominant; C3, inflammatory; C4, lymphocyte depleted; C6, TGF-β dominant. **(C)** Heatmap of Spearman’s correlation between transcriptome profiles, risk scores, and immune infiltration. Only statistically significant correlations are shown (*P <*0.05). **(D)** Spearman’s correlation of Th2/Th17 infiltration, M0/M1 macrophage infiltration, and the glycosylation-based risk score. **(E, F)** Different relative proportions of immune cells in different groups. **(G)** Scores of the five distinct immune indices between the low- and high-risk groups. *P <0.05; **P <0.01; ***P < 0.001, ns, not significant.

Immune suppression drives tumor evolution toward a stem cell-like phenotype. This study assessed the correlation between risk classification and immune molecular subtype and found that C4 (lymphocyte-depleted) was the most common subtype (**Figure 7A**). A higher proportion of C1 and C2 subtypes, and a lower level of the C3 subtype, were associated with a poorer prognosis (*P <*0.0001; **Figure 7B**). The correlation between immune cell infiltration and glycosylation-related gene expression also varied (**Figure 7C**). In the TCGA-LIHC cohort, correlation analyses further revealed a clear positive association between risk scores and infiltrating Th2 cells (R = 0.33; *P* = 3.6e−10) and M0 macrophages (R = 0.36; *P* = 1.2e−12). In contrast, both Th17 cells (R = −0.31; *P* = 3e−9) and M1 macrophages (R = −0.13; *P* = 0.018) were negatively associated with risk scores (**Figure 7D**). Similar results were also shown in the ICGC-LIRI-JP cohort (**Supplementary Figure 4A**). Differences in immune cells were also evaluated between the low- and high-risk groups in the TCGA-LIHC and ICGC-LIRI-JP cohorts. While aDCs and macrophages were significantly increased in the high-risk groups in both the TCGA-LIHC and ICGC-LIRI-JP cohorts, B cells, CD8^+^ T cells, mast cells, neutrophils, NK cells, and TIL were reduced in the high-risk score group in the TCGA-LIHC cohort (**Figures 7E, F**). Meanwhile, only NK cells were reduced in the high-risk group in the ICGC-LIRI-JP cohort (**Supplementary Figures 4B, C**). Some immune-related scores were also assessed in the low- and high-risk groups (**Figure 7G**). Except for a striking negative association between the IFN-γ response score and the high-risk subgroup, other scores were significantly elevated in the high-risk group. These results indicated that high-risk patients had a distinct stemness phenotype associated with the accumulation of immune-suppressive cells.

### Validation of the Prognostic Signature and Establishment of a Novel Nomogram

**Figure 8:**
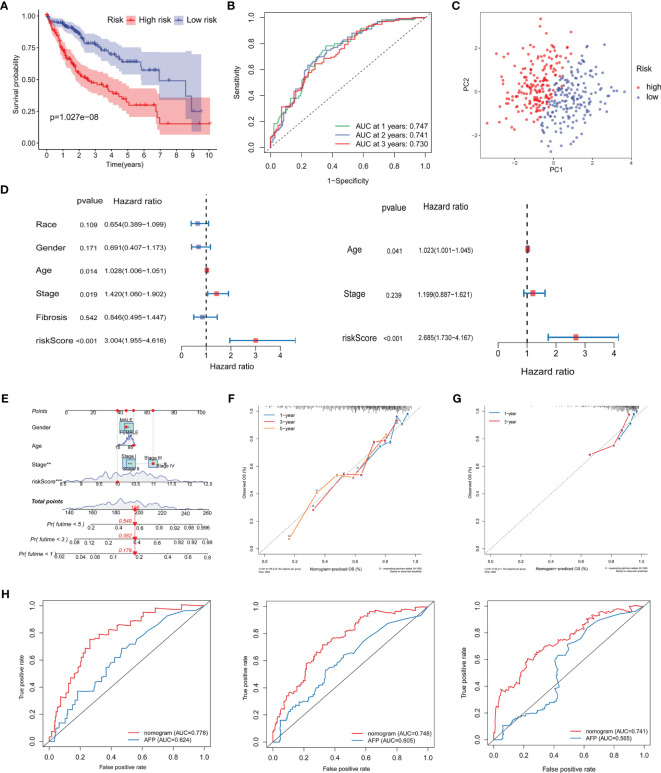
Validation of the prognostic signature and establishment of a novel nomogram. **(A)** Kaplan–Meier curve analysis of the low- and high-risk groups. **(B)** ROC curve showing the prognostic risk model. **(C)** PCA plot of TCGA-LIHC patients in different risk groups. **(D)** Univariate and multivariate independent prognostic analysis of clinical features and risk scores. **(E)** Nomogram for survival prediction in TCGA-LIHC. **(F, G)** Nomogram calibration curves of 1-, 3-, and 5-year survival probabilities in TCGA-LIHC and ICGC-LIRI-JP, respectively. **(H)** ROC curves of the nomogram and AFP for the survival prediction at 1-, 3-, and 5-years. ROC, receiver operating characteristic; AUC, areas under the receiver operating characteristic curve; PCA, Principal component analysis; AFP, alpha-fetoprotein; OS overall survival.

HCC patients in the high-risk group had a significantly poorer prognosis and lower OS than those in the low-risk group (**Figure 8A**; **Supplementary Figure 5A**). The area under the ROC curve (AUC) of the risk score in predicting 1-, 2-, and 3-year survival was 0.747, 0.741, and 0.730, respectively, in the TCGA dataset and 0.672, 0.642, and 0.647, respectively, in the ICGC dataset (**Figure 8B**; **Supplementary Figure 5B**). Compared with the risk subgroups, whole gene expression patterns were separated into two dispersion directions in both the ICGC and TCGA (**Figure 8C**; **Supplementary Figure 5C**). Race, gender, age, stage, fibrosis, and risk score were then included in univariate and multivariate Cox regression analyses. Notably, risk score was found to be an independent risk factor (HR >1, *P <*0.001) (**Figure 8D**). To further verify the prognostic value of the risk score, a nomogram that included gender, age, stage, and risk score was designed to illustrate patient survival more intuitively (**Figure 8E**). The calibration curves evaluated the predictive power of the nomogram at 1, 3, and 5 years, and the C-indexes were 0.704, 0.705, and 0.703, respectively (**Figure 8F**). The ICGC dataset was used to validate the nomogram and showed better discrimination and calibration ability, with C-indexes of 0.737, 0.739, and 0.739, respectively (**Figure 8G**). The AUC values of the nomogram at 1, 3, and 5 years reached 0.778, 0.748, and 0.741, respectively, which were better than those of the traditional HCC marker, AFP (**Figure 8H**).

### Drug Sensitivity Analysis for the Five Glycosylation-Related Prognostic Genes

**Figure 9:**
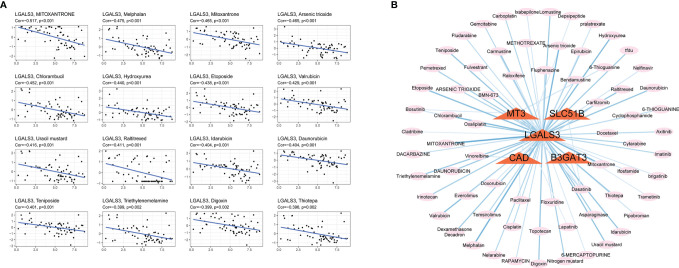
Screening for sensitive drugs. **(A)** The top 16 most relevant negative correlations. **(B)** The network between five genes and sensitive drugs with negative IC50s (R<−0.25, *P <*0.05). Stronger correlations are represented by a thick dark blue line.

Glycosylation provides a range of potential targets for therapeutic intervention. However, potential drugs are still in clinical trials. Thus, the drug sensitivity of five selected glycosylation-related genes across diverse human cancer cell lines was further analyzed (correlation coefficient |R| >0.25, *P <*0.05). Consequently, 84 sensitive drugs were screened for abnormal glycosylation during HCC. Carfilzomib had the most obvious correlation with CAD (R = −0.341, P = 0.008) and mitoxantrone had the most obvious correlation with LGALS3 (R = −0.517, P = 2.36e−5). Dasatinib is associated with several genes. The top 16 strongest negative correlations between gene expression and IC50 are shown in **Figure 9A**. The link between the genes and sensitive drugs with a negative correlation of IC50 is shown in **Figure 9B**.

## Discussion

HCC involves complex architecture and heterogeneity and lacks effective individualized treatment targets (30). LCSCs with a specific phenotype are believed to promote HCC relapse, metastasis, and chemoresistance. Recent studies have shown that aberrant glycosylation of signaling pathways and LCSC markers directly impacts key processes that maintain cell survival, self-renewal, and extravasation properties (31–33). This study focused on the importance of glycosylation in promoting the stemness of HCC by assessing alterations in somatic mutations, immune cell infiltration, and clinical characteristics. Potential target drugs that could be used to improve HCC prognosis were selected.

To our knowledge, this is the first integrated multi-omics study in which the association between glycosylation and stemness was shown in HCC patients using bulk and single-cell RNA-sequencing data. A glycosylation-related prognostic signature was constructed consisting of five genes: CAD, B3GAT3, SLC51B, LGALS3, and MT3, using univariate, stepwise, and multivariate Cox regression analysis. Patients were then divided into low- and high-risk subgroups. Gene expression profiles of the high-risk group correlated positively with the upregulation of CSC markers, CD24, CD44, CD20, FOXM1, and EpCAM, and significant enrichment of other ESC signatures, indicating that these were tumor-promoting targets. Several studies have shown that stemness is associated with genetic mutations, epigenetic changes, and differences in the tumor microenvironment (34, 35). Similarly, our study found that the high-risk group had unique somatic mutations, CNVs, and immune patterns as well as enriched stem cell-like characteristics and high levels of gene expression. Clinical information was then combined with the prognostic signature to construct a calibrated nomogram. Finally, the sensitivity of glycosylation-related genes to particular drugs was evaluated, providing novel insight into tumor treatments and the prevention of drug resistance. Among the drug candidates, carfilzomib showed the most obvious correlation with CAD.

The five glycosylation-related genes were identified as significant in HCC. CAD is a multifunctional protein that takes part in *de novo* pyrimidine nucleotide synthesis, protein glycosylation, and phospholipid biosynthesis in mammals (12). This protein must initiate the di-(UDP)-dependent glycosylation process by producing UDP. UDP-N-acetylglucosamine (UDP-GlcNAc), for example, serves as an essential sugar donor substrate for O-GlcNAc, which is involved in a central intracellular protein modification in diverse metabolism, signaling, and disease processes (36). A recent study by Ching-Yu et al. (37) showed that UDP overexpression significantly promoted human hepatoma cell proliferation both *in vitro* and *in vivo*. UDP-13C-glucose flux is also responsible for metabolic reprogramming and the expression of key stemness genes in CSCs (38). The expression of LGALS3, a siglec-9 ligand in cancer cells, correlates with tumor progression and increased expression of β-catenin and CSC markers induced through Wnt signaling (39, 40). LGALS3 also helps maintain LSCS stemness, expansion, and aggressiveness and may thus serve as a target for HCC treatment (41). B3GAT3 is a glucuronosyltransferase involved in glycosylation and the proliferation and metastasis of HCC tissues and cells (42). SLC51B is involved in the intestinal reclamation of bile acids and steroids and eicosanoid metabolism, promoting liver cancer cell proliferation and suppressing apoptosis, which is associated with stemness (43). MT3 is a member of the metallothionein family that regulates protein glycosylation and is closely linked to HCC progression. Studies indicate that MT overexpression may induce tumor cell differentiation (44, 45), but the mechanism will require further investigation. This study is consistent with the previously reported findings while also furthering our understanding of the relationship between LCSCs and glycosylation.

The production and activation of LCSCs may be induced by a series of gene mutations in normal stem or progenitor cells that disrupt cell metabolism, immune escape, and drug resistance. CNV is a hallmark of amplifications or deletions in the cancer genome, which can inactivate tumor suppressor genes, induce high oncogene expression, and increase stemness. A recent study of CNV showed that increases in the E2f1 or E2f3b loci promoted spontaneous HCC, whereas decreases in these loci suppressed HCC (46). In a genome-wide analysis, TRIM35 was shown as a novel tumor suppressor (47), and COL4A1 on the 13q34 locus was a frequent amplification target, a finding consistent with our results (48). Our study found that the RB1 deletion was most significant in the high-risk group, which supports prior research by Sung-Min et al. (49).

Stemness is significantly associated with immunosuppression in the immune microenvironment. As a result, this study also compared the proportions of immune cells in the high- and low-risk score groups and their gene expression profiles. Interestingly, cancer stem cell-like characteristics were enriched in the high-risk groups. To characterize intratumoral immune states, HCC samples were clustered into six subtypes using definitions developed by Thorsson et al. (29). The C4 subtype, associated with an imbalanced ratio of Th2/Th17 and M0/M1 cells, was the most common. Th2-related cytokines such as IL-4, IL-5, and IL-10 are involved in stimulating B cell proliferation and mediating humoral immunity. Some studies indicate that Th2 cells induce HCC progression (50). Indeed, Th2 cytokines facilitate tumor escape by inhibiting Th1 cytokine production (51) and furthering the progression of liver damage. However, IL-17 production by Th17 cells is generally associated with higher cancer survival (52). After transcatheter arterial embolization (TAE), the number of Th17 cells in the tumor microenvironment increases significantly (53). The Th2/Th17 cytokine profile is unbalanced during HCC and the shift towards Th17 cells can promote anti-tumor effects. Macrophages are key antigen-presenting cells of the innate immune system, and different macrophage phenotypes have distinct functions in regulating the tumor microenvironment. In particular, M1 macrophages are involved in suppressing tumor growth and inducing liver tumor regression. Guerra et al. (54) found that hydrogel-embedded M1 macrophages induced apoptosis of HCC cells and promoted tumor regression. Localization of activated M1 macrophages may be a new novel HCC treatment strategy. Our study also found significant enrichment in intratumor heterogeneity, TGF-β mediated signaling, proliferation, and a cancer stemness phenotype in the high-risk group, while IFN signaling was increased in the low-risk group. Consistent with these results, TGF-β-mediated signaling has been strongly linked to a cancer stem-like phenotype in tumors (55), while IFN signaling has been negatively correlated with stemness and HCC progression (56, 57).

Current chemotherapy drugs remain unable to significantly promote the long-term survival of HCC patients. Thus, this study screened potential target drugs using the Cell-Miner database. Carfilzomib, a second-generation proteasome inhibitor, had the strongest correlation with CAD. This drug exhibits an anti-tumor effect by inhibiting MAPK signaling (58). CAD activation requires MAPK, so it is possible that carfilzomib indirectly inhibits CAD by suppressing MAPK signaling and promoting an anti-tumor response. Mitoxantrone, an antineoplastic drug developed in the 1980s, had the strongest correlation with genes. A recent study found that mitoxantrone inhibits HCC growth and proliferation by inducing autophagy (59). In the mitoxantrone-resistant cell line, LGALS3 was detected with an abundance ratio well above two (60). Dasatinib, a tyrosine kinase inhibitor that exerts anti-tumor effects by inhibiting glucuronosyltransferase, is associated with the expression of several genes (61). SLC51B is a subtype of the human solute carrier (SLC) and correlates negatively with drug sensitivity (62). MT3 is also involved in SLC synthesis and is negatively associated with Dasatinib. BAGAT3 is an important type of glucuronosyltransferase. However, whether these drugs could be used to treat abnormal glycosylation requires further study.

Glycosylation is a promising target for tumor treatment. However, there are some limitations that should be addressed in our study. First, few studies have assessed the relationship between stemness and glycosylation, especially those involving the development of HCC. This study relied heavily on TCGA, GEO, and ICGC data and lacked experimental evidence. Thus, we could not determine whether the selected genes have corresponding roles in glycosylation pathways. Further functional experiments are necessary to define the molecular mechanisms. Second, CNVs were not available in the ICGC database. A larger CNV sample size would help ensure the reliability of the study. Third, the mechanism of potentially sensitive drugs in abnormal glycosylation during HCC remains unknown and requires further study.

## Conclusions

In conclusion, this study proposed a prognostic signature founded on glycosylation-related gene expression and classified HCC samples into low- and high-risk subgroups. According to bulk and single-cell RNA-sequencing, the high-risk group was most likely to have a stem-like phenotype. This was verified by assessing somatic mutations, CNVs, immune infiltration, and clinical characteristics. These findings will help inform future studies on the relationship between glycosylation and HCC stemness.

## Data Availability Statement

The original contributions presented in the study are included in the article/**Supplementary Material**. Further inquiries can be directed to the corresponding author.

## Ethics Statement

The studies involving human participants were reviewed and approved by the Ethics Committee of Tianjin Second People’s Hospital. The patients/participants provided their written informed consent to participate in this study.

## Author Contributions

All authors contributed to the study conception and design. PL and QZ contributed equally to this study. PL and QZ designed and wrote the manuscript. PL and QZ screened the literature and collected data. PL and QZ were responsible for pictures and statistics. PL and QZ polished the article. JL critically revised the manuscript. All authors listed have made a substantial, direct, and intellectual contribution to the work and approved it for publication.

## Funding

This work was funded by the Natural Science Foundation of Tianjin City (20JCYBJC01150); the Tianjin Health Science and Technology Project (Nos. TJWJ2021QN063, TJWJ2021ZD010, and TJWJ2021MS034), and the Tianjin Key Medical Discipline (Specialty) Construction Project.

## Conflict of Interest

The authors declare that the research was conducted in the absence of any commercial or financial relationships that could be construed as a potential conflict of interest.

## Publisher’s Note

All claims expressed in this article are solely those of the authors and do not necessarily represent those of their affiliated organizations, or those of the publisher, the editors and the reviewers. Any product that may be evaluated in this article, or claim that may be made by its manufacturer, is not guaranteed or endorsed by the publisher.

## Supplementary Material

Supplementary Table 1 Glycosylation-related pathways and genes retrieved from MSigDB. MSigDB, Molecular signatures Database.

Supplementary Table 2 Primer and specific siRNA sequences were used in this study. siRNA, small interfering RNA.

Supplementary Figure 1 The representative immunohistochemistry of the five glycosylation-related genes in normal and HCC tissues downloaded from HPA. HPA, The Human Protein Atlas.

Supplementary Figure 2 Relationship between gene expression profiles and HCC stemness using HCC bulk data from ICGC-LIRI-JP. **(A)** Heatmap of Spearman’s correlation results of the gene expression profiles and four distinct stemness indices (Ben-Porath signature, Wong signature, Bhattacharya signature, and mRNA-si). A darker color represents a stronger correlation. **(B)** Correlation between the CSC markers, CD24, CD44, CD20, FOXM1, and EpCAM, and the gene expression profiles. **(C)** Different scores of the four distinct stemness indices between the low- and high-risk groups. **(D)** The transcriptome profiles of high-risk HCC patients were significantly enriched with stemness markers. **(E)** Among the high-risk patients, the frequency of higher stages and grades was more significantly elevated. Tumor stages and grades were color-coded as shown in the legend. NES, normalized enrichment score; CSC, cancer stem cells.

Supplementary Figure 3 The influence of the remaining four genes on the stemness phenotype of HCC. **(A)** Differential expression in HCC and normal cells. **(B)** Significant decrease in stemness-related markers, CD24, CD44, CD20, FOXM1, and EpCAM, after SLC51B knockdown in HepG2 and Huh7 cells. **(C)** Significant decrease in stemness-related markers, CD24, CD44, CD20, FOXM1, and EpCAM, after LGALS3 knockdown in HepG2 and Huh7 cells. **(D)** Significant decrease in stemness-related markers, CD24, CD44, CD20, FOXM1, and EpCAM, after B3GAT3 knockdown in HepG2 and Huh7 cells. **(E)** Significant decrease in stemness-related markers, CD24, CD44, CD20, FOXM1, and EpCAM, after MT3 knockdown in HepG2 and Huh7 cells. *** *P* < 0.001.

Supplementary Figure 4 The immune landscape of HCC tumors in ICGC-LIRI-JP. **(A)** Spearman’s correlation of Th2/Th17 infiltration, M0/M1 macrophage infiltration, and the glycosylation-based risk score. **(B, C)** Different relative proportions of immune cells in different groups. **P <*0.05; ***P <*0.01; *** *P <*0.001.

Supplementary Figure 5 Validation of the prognostic signature in ICGC-LIRI-JP. **(A)** Kaplan-Meier curve analysis of the low- and high-risk groups. **(B)** ROC curve showing the prognostic risk model. **(C)** PCA plot of TCGA-LIHC patients in different risk groups. ROC, receiver operating characteristic; PCA, principal component analysis.
