# Supplementary figures and images for: Integrated Multi-Omics Data Analysis Reveals Associations Between Glycosylation and Stemness in Hepatocellular Carcinoma
Source: Front Oncol. 2022 Jun 23;12:913432. doi: 10.3389/fonc.2022.913432 (PMC9259879; doi:10.3389/fonc.2022.913432)

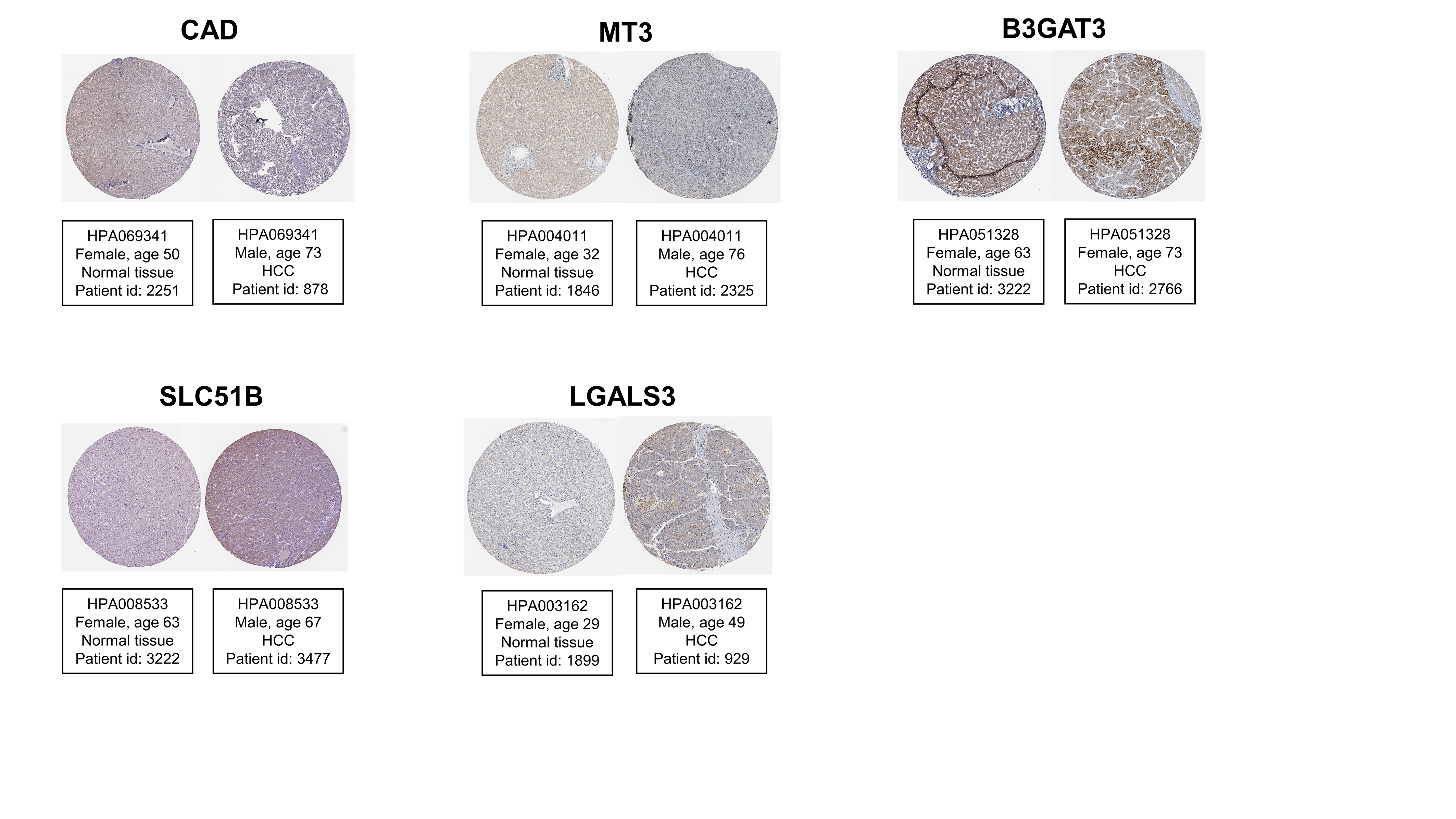

Supplement: Supplementary Figure 1 — The representative immunohistochemistry of the five glycosylation-related genes in normal and HCC tissues downloaded from HPA. HPA, The Human Protein Atlas. [file Image_1.tif]

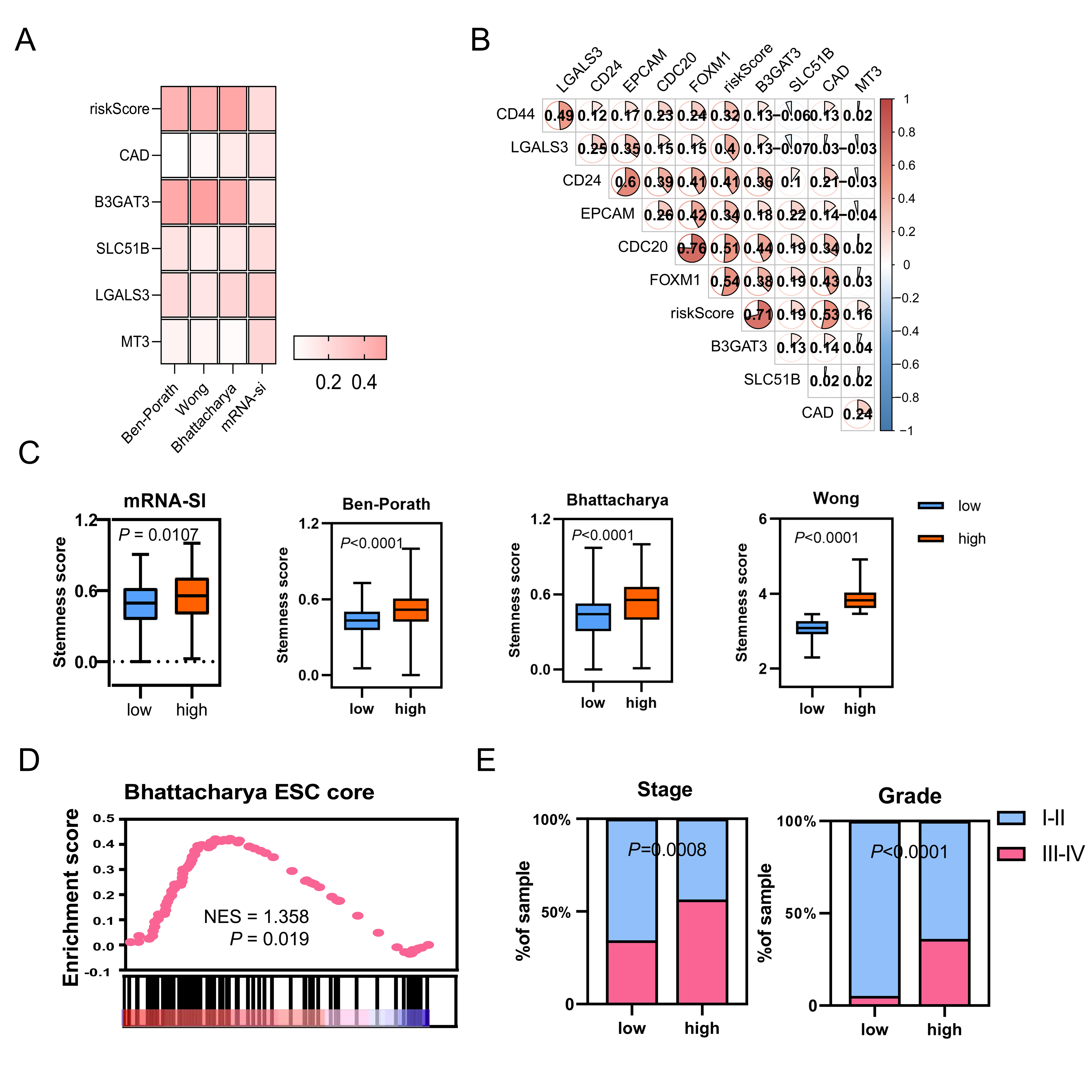

Supplement: Supplementary Figure 2 — Relationship between gene expression profiles and HCC stemness using HCC bulk data from ICGC-LIRI-JP. (A) Heatmap of Spearman’s correlation results of the gene expression profiles and four distinct stemness indices (Ben-Porath signature, Wong signature, Bhattacharya signature, and mRNA-si). A darker color represents a stronger correlation. (B) Correlation between the CSC markers, CD24, CD44, CD20, FOXM1, and EpCAM, and the gene expression profiles. (C) Different scores of the four distinct stemness indices between the low- and high-risk groups. (D) The transcriptome profiles of high-risk HCC patients were significantly enriched with stemness markers. (E) Among the high-risk patients, the frequency of higher stages and grades was more significantly elevated. Tumor stages and grades were color-coded as shown in the legend. NES, normalized enrichment score; CSC, cancer stem cells. [file Image_2.tif]

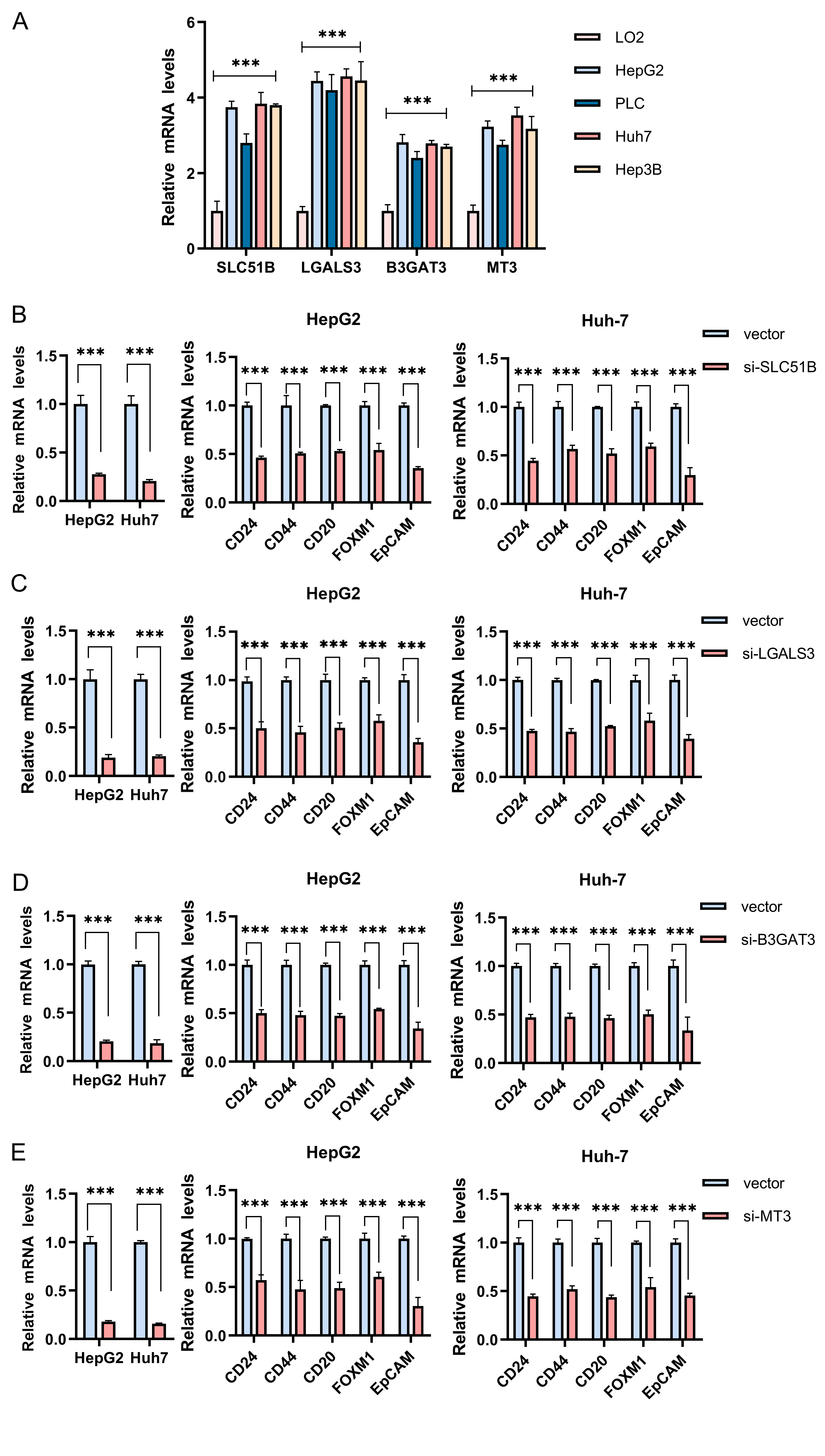

Supplement: Supplementary Figure 3 — The influence of the remaining four genes on the stemness phenotype of HCC. (A) Differential expression in HCC and normal cells. (B) Significant decrease in stemness-related markers, CD24, CD44, CD20, FOXM1, and EpCAM, after SLC51B knockdown in HepG2 and Huh7 cells. (C) Significant decrease in stemness-related markers, CD24, CD44, CD20, FOXM1, and EpCAM, after LGALS3 knockdown in HepG2 and Huh7 cells. (D) Significant decrease in stemness-related markers, CD24, CD44, CD20, FOXM1, and EpCAM, after B3GAT3 knockdown in HepG2 and Huh7 cells. (E) Significant decrease in stemness-related markers, CD24, CD44, CD20, FOXM1, and EpCAM, after MT3 knockdown in HepG2 and Huh7 cells. *** P < 0.001. [file Image_3.tif]

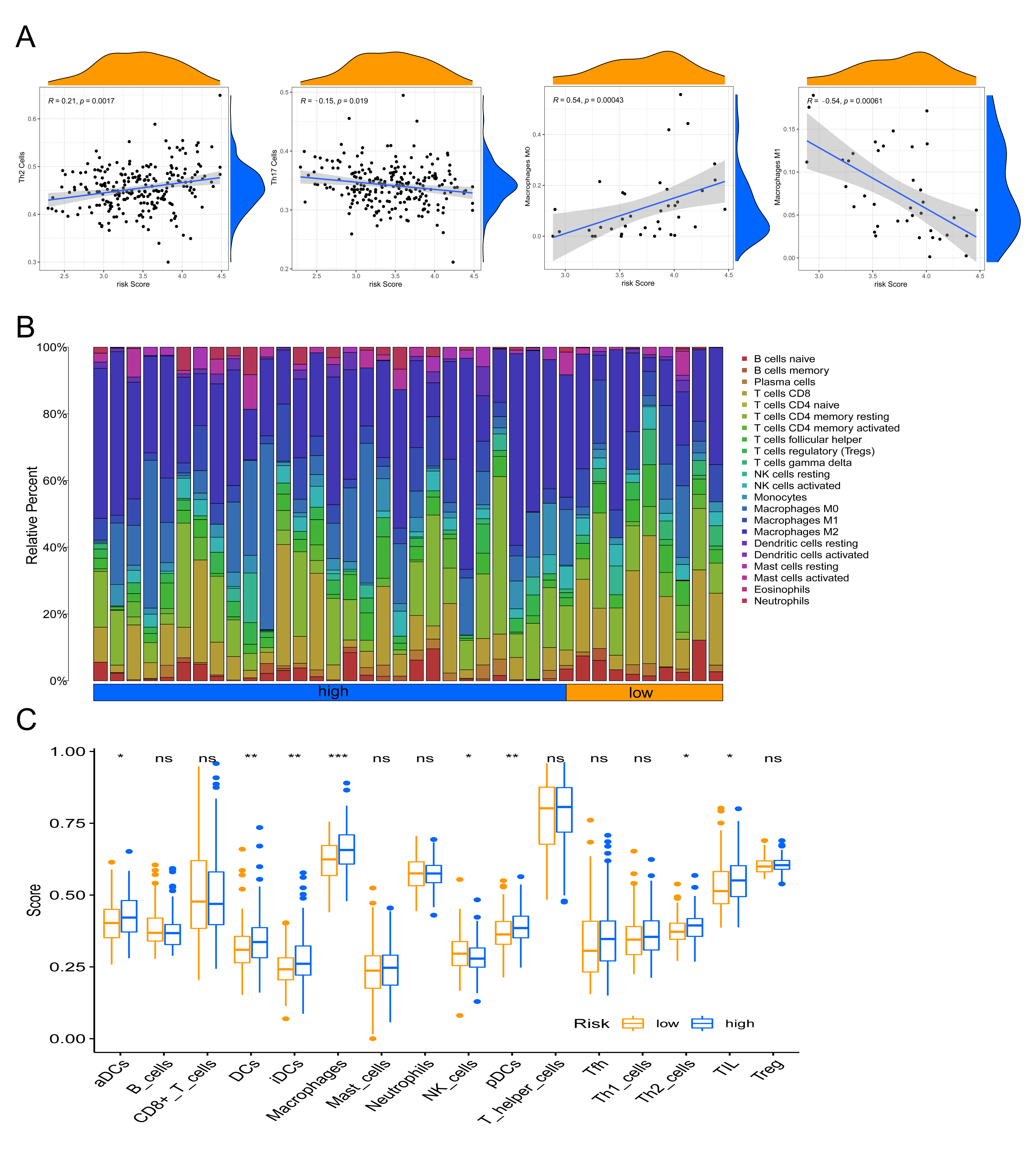

Supplement: Supplementary Figure 4 — The immune landscape of HCC tumors in ICGC-LIRI-JP. (A) Spearman’s correlation of Th2/Th17 infiltration, M0/M1 macrophage infiltration, and the glycosylation-based risk score. (B, C) Different relative proportions of immune cells in different groups. *P <0.05; **P <0.01; *** P <0.001. [file Image_4.tif]

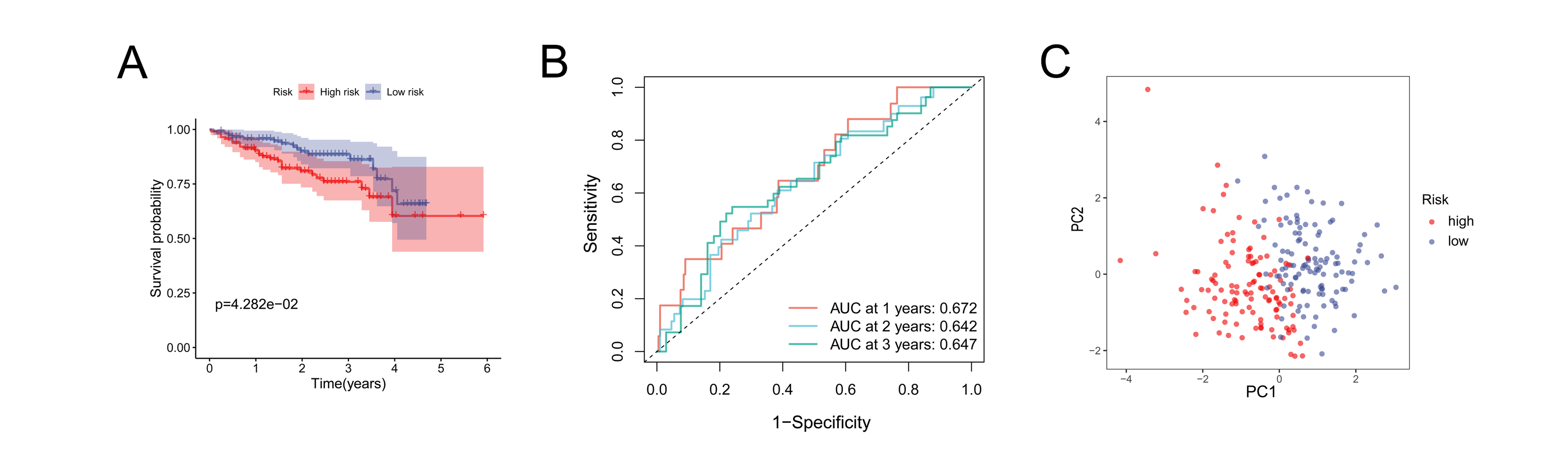

Supplement: Supplementary Figure 5 — Validation of the prognostic signature in ICGC-LIRI-JP. (A) Kaplan-Meier curve analysis of the low- and high-risk groups. (B) ROC curve showing the prognostic risk model. (C) PCA plot of TCGA-LIHC patients in different risk groups. ROC, receiver operating characteristic; PCA, principal component analysis. [file Image_5.tif]
